# Genetic association of the rs17782313 polymorphism with antipsychotic-induced weight gain

**DOI:** 10.1007/s00213-023-06331-9

**Published:** 2023-02-09

**Authors:** Korbinian Felix Schreyer, Stefan Leucht, Stephan Heres, Werner Steimer

**Affiliations:** 1 Department of Clinical Chemistry and Pathobiochemistry, School of Medicine, Technical University of Munich, Ismaninger Str. 22, 81675 Munich, Germany; 2 Department of Psychiatry and Psychotherapy, School of Medicine, Technical University of Munich, Ismaninger Str. 22, 81675 Munich, Germany; 3 kbo-Klinik für Psychiatrie und Psychotherapie Nord – Schwabing, Kölner Platz 1, 80804 Munich, Germany

**Keywords:** Amisulpride, Antipsychotic treatment, Human melanocortin four receptor gene (*MC4R*), Olanzapine, Pharmacogenetics, rs17782313, Second generation antipsychotics, Weight gain

## Abstract

**Rationale:**

Weight gain is a frequent side effect of treatment with SGAs (second-generation antipsychotics) and a leading cause for nonadherence. Several candidate genes have been identified that could influence the amount of AIWG (antipsychotic-induced weight gain). The polymorphism rs17782313 near the *MC4R* (human melanocortin 4 receptor gene) was strongly associated with obesity in a large scale GWAS (genome wide association study), yet previous studies investigating its impact on AIWG did not lead to a definite conclusion regarding its effect. In particular, they were all relatively short and had a naturalistic design.

**Objective:**

We therefore examined the influence of the rs17782313 polymorphism on SGA-related weight gain.

**Methods:**

Participants of a multicenter randomized, controlled, double-blind study comparing two treatment strategies in individuals with schizophrenia or schizoaffective disorder were genotyped using a rapid-cycle polymerase chain reaction. Up to 252 individuals completed the first 2 weeks (phase I), 212 the entire 8 weeks (hence ‘completers’). Patients received either amisulpride or olanzapine or both consecutively. Thirty-seven had their first episode. Weight gain occurring in different genotypes was statistically compared and confounding factors were adjusted by stepwise multiple linear regression. A correction for multiple testing was included.

**Results:**

Within 212 ‘completers’, carriers of the C allele had a higher absolute weight gain than those homozygous for the T allele (2.6 kg vs. 1.2 kg), though this observation was not significant (*P* = 0.063). In the amisulpride subpopulation, this association appeared stronger and reached significance (2.5 kg vs. 0.7 kg, *P* = 0.043), though failed to remain significant after correction for multiple testing. A stepwise multiple linear regression showed a significant association in both the whole study population (*P* < 0.001) and the amisulpride subpopulation (*P* < 0.001).

**Conclusion:**

Our results indicate that the rs17782313 polymorphism might influence antipsychotic-induced weight gain and therefore confirm some of the earlier conclusions.

**Supplementary Information:**

The online version contains supplementary material available at 10.1007/s00213-023-06331-9.

## Introduction

Schizophrenia is a chronic, psychotic mental disease that affects approximately one percent of people. The time of onset is typically in late adolescence or early adulthood and regularly continues through the whole life (Freedman 2003). It presents itself as a spectrum of disorders with positive, negative, cognitive, and affective symptoms. Its most common form, paranoid schizophrenia, is often characterized by paranoia and auditory delusions (Insel 2010).

Pharmacological treatment was introduced more than half a century ago with the discovery of D_2_ (dopamine 2 receptor)-antagonists such as chlorpromazine and haloperidol. It later shifted to SGAs (second-generation antipsychotics) that owing to their lower affinity to the dopamine receptor and a higher affinity to serotonin and norepinephrine receptors tend to show better improvement in negative symptoms and fewer extrapyramidal side effects. However, this alleged advantage is often bought at the price of metabolic alterations such as weight gain, hyperglycemia, or dyslipidemia (Allison and Casey 2001; Miyamoto et al. 2005), being a leading cause for nonadherence (Lieberman et al. 2005; Weiden et al. 2004). These adverse effects do not equally affect every patient though. While the substance is known to greatly influence the amount of weight gain (clozapine and olanzapine rank among the least favorable), genetic variation has also been suggested to play an important role (Shams and Müller 2014).

One of the leading causative genes regarding weight gain itself is *MC4R* (human melanocortin receptor gene). As more than 130 *MC4R* mutations have been detected, of which many lead to a loss of function (Fan and Tao 2009), the common SNP (single nucleotide polymorphism) rs17782313 showed the strongest association with BMI (body mass index) in a large-scale GWAS (genome wide association study) (Loos et al. 2008), which analyzed 16,876 individuals of European descent. The findings were confirmed in 60,352 adults, 5988 children, and 660 German families. The overall combined per-allele effect on BMI was 0.049 (0.037–0.061/Z-score units, *P* = 2.8 × 10^15^) in 77,228 genotyped adults.

A GWAS conducted later reported 20 SNPs near the *MCR4* that exceeded a certain statistical threshold when assessing weight gain after 12 weeks of antipsychotic treatment in pediatric patients, underlining the possible relevance of mutations near the *MC4R* in extreme SGA-induced weight gain (Malhotra et al. 2012). The rs17782313 is located in close proximity to one of these 20 SNPs that can be found approximately 190 kilobases downstream from the *MC4R*, a locus overlapping the region identified by the GWAS from above.

So far, few studies have investigated the influence of the rs17782313 polymorphism on antipsychotic-induced weight gain and produced not entirely consistent results. Czerwensky et al. (2013) reported a significant influence of the polymorphism on weight gain in a naturalistic study with 345 patients after 4 weeks of treatment with various second-generation antipsychotics and showed similar results in a first-episode subpopulation and a subpopulation not receiving co-medication known to induce weight gain. Chowdhury et al. (2013) analyzed 224 patients that received antipsychotic treatment over up to 6 weeks and could not reveal a significant association, yet their analysis yielded a trend for higher AIWG and the C allele in a European sub-sample treated with clozapine or olanzapine. A meta-analysis published in 2016 only listed those two studies for this particular SNP. The combined effect showed a tendency, though lacked significance (Zhang et al. 2016). Zhang et al. (2019) could not find a significant association of the rs17782313 polymorphism and BMI percentage change in neither a total population of 1991 Han Chinese patients treated for schizophrenia for 6 weeks nor in subgroups with drug-naïve or medicated subjects.

After more than a decade since the discovery of rs17782313 as a strong mediator in obesity and nearly 10 years since the first investigation of its influence in AIWG, there remains a need for more studies to clarify the effect it has on SGA-related weight gain and create a basis for future meta-analyses on this matter. Thus, we investigated the influence of rs17782313 on weight gain in 252 patients that took part in a multicenter randomized, controlled, double-blind study comparing two treatment strategies for acute schizophrenia or schizoaffective disorder.

## Materials and methods

### Study design

Patients aged between 18 and 65 who suffered from schizophrenia, schizoaffective disorder, or schizophreniform disorder were recruited as part of a multi-center study (Heres et al. 2016). At the beginning of the 8-week lasting trial, patients were randomly assigned to two treatment groups, each one receiving amisulpride or olanzapine. After 2 weeks of treatment (resembling phase I), those individuals experiencing a ‘non response’ were randomized again, resulting in either a switch to the other medication or a continuation with the originally assigned drug for another 6 weeks (phase II). All ‘responders’ continued their medication throughout phase II. Therefore, patients received either amisulpride or olanzapine for 8 weeks, or switched after 2 weeks from one to the other. One site was excluded due to misconduct in a later, independent trial. Complete data regarding baseline weight and weight gain and consent to genetic testing was available for 252 subjects for the first 2 weeks (phase I) respectively for the entire trial — due to drop outs — for 212 individuals (hereinafter referred to as completers). No additional antipsychotics, mood stabilizers or recently initiated antidepressants were given. Rescue medication for symptomatic treatment of agitation, sleep disturbances or side effects were benzodiazepines and Z-drugs. The design of the study was double-blind. Neither the treating physicians nor the patients knew which study drug was assigned to them in phase I, nor if they switched to the other substance in phase II or maintained the treatment they started with. Group allocation and distribution of the medication were conducted by the pharmacy of the University of Mainz, which did not take part in conducting the study or in analyzing the results. More details can be found in Heres et al. (2016).

### Genotyping

We genotyped the rs17782313 polymorphism, a T to C transition located 188 kb downstream from the *MC4R* with a minor allele frequency of ~23.1% in Europeans (Phan et al. 2020). DNA was prepared using the QIAmp® DNA Blood Mini Kit. We performed a rapid-cycle polymerase chain reaction (Czerwensky et al. 2013) on the LightCycler® 2.0 (Roche, Penzberg, Germany) as described elsewhere (Bui and Liu 2009; Popp et al. 2003).

### Statistics

We analyzed weight gain and BMI increase occurring both during the first 2 weeks (phase I) and weight gain during the entire 8 weeks. This was due to the design of the study (the ‘switch’ in some patients’ medication after 2 weeks) and, as expected, discontinuations of some individuals. For both time periods, we at first looked at all patients combined and in a second step at individuals that received only either one of the SGAs. In addition, we analyzed a ‘first episode’ subpopulation with *n* = 37 individuals, though baseline height and therefore baseline BMI of one individual was missing. Relative weight gain in % and relative BMI increase in % are mathematically equal, we therefore reported only on the former. To sum up, in the Results section we (1) reported on Hardy-Weinberg equilibrium and (2) baseline characteristics for (2a) all taking part in phase I, for (2b) completers of the trial, and (2c) first episode patients. Then we focused on (3) weight gain occurring within the first 2 weeks in (3a) all medication arms, (3b) those treated with amisulpride, (3c) those receiving olanzapine, and (3d) in first episode patients. In (4) we analyzed weight gain in ‘completers’ of the trial (4a) regardless of medication (hence including those having switched), (4b) in individuals receiving amisulpride only, (4c) in patients treated with olanzapine only, and (4d) in first episode patients. Finally, in (5), we corrected our results for multiple comparisons (for method see below).

Statistical analysis was carried out using IBM SPSS Statistics for Windows, version 21.0 (IBM Corp., Armonk, N.Y., USA). We checked raw data for plausibility, then calculated different variables for further analysis, for example for each individual patient i:

$${\mathrm{BMI}}_{\mathrm i}\left[\frac{\mathrm{kg}}{\mathrm m^2}\right]=\frac{\mathrm{Baseline}\;{\mathrm{Body}\;\mathrm{Weight}}_{\mathrm i}\;\left[\mathrm{kg}\right]}{{\mathrm{Height}}_{\mathrm i}\;\left[\mathrm m\right]^2}$$ and $${\mathrm{Age}}_{\mathrm i}\;=\;\mathrm{Year}\;\mathrm{of}\;{\mathrm{participation}}_{\mathrm i}\;-\;\mathrm{Year}\;\mathrm{of}\;{\mathrm{Birth}}_{\mathrm i}$$

Two-tailed *P* = 0.05 were considered to be statistically significant (α = 0.05). To check if the population was in Hardy-Weinberg equilibrium, the chi-square test was used. Reference data was retrieved from the Allele Frequency Aggregator (ALFA) (Phan et al. 2020) — European sample, release version 20201027095038, minor allele frequency MAF_c_ = 0.231114. Expected frequencies n_TT_, n_TC_, and n_CC_ were calculated f_TT_ = (1 − MAF_c_)^2^ × (n_TT_ + n_TC_ + n_CC_) etcetera in accordance with Hardy-Weinberg law. Normal distribution was determined graphically and through the Kolmogorov-Smirnov and Shapiro-Wilk test (see Supplementary Table 1). Absolute weight gain, relative weight gain, absolute increase in BMI, and relative increase in BMI were compared. In case of normal distribution, we used two-tailed *T*-test (or Welch’s *T*-test if equal variances were rejected in Levene’s test) for comparison of two groups and analysis of variance for three groups. If normal distribution was violated, we used the Mann-Whitney *U* test for pairwise comparisons and the Kruskal-Wallis test for groupwise comparisons (see Supplementary Table 1 for normal distribution of variables). To adjust for confounding effects on relative weight gain in % (as done in Czerwensky et al. (2013)) and to calculate combined effects, stepwise multiple linear regression analyses were calculated. Possible covariates were entered stepwise into the model and parameters were included when the 95% confidence interval of their standard error did not include zero and the objective function value fell by more than 3.94, equaling a significance (probability) of the *F* value of 0.05. Although our approach was of exploratory nature, a correction for multiple testing was included to evaluate robustness of statistical findings. Our main research question was the influence of the rs17782313 on AIWG. *P*-values of baseline characteristics were not corrected as we think they were not of interest in this analysis and can be seen as a mean to find confounding variables. As weight gain and BMI gain can be interpreted as two sides of the same coin and specific differences between them were not our focus, we corrected *P*-values of weight gain within 2 weeks and weight gain within 8 weeks for both the comparison of TT vs TC vs CC and TT vs Cx in all medication groups, the first episode subpopulation, and the MC4R genotype as a factor in the multiple linear regression analyses for multiple comparison by Holm’s procedure (Holm 1979; Ludbrook 1998). Thus, m = 25 *P*-values were arranged in ascending order for all hypotheses of interest. All P_k_, while k was the *P*-value’s index and α = 0.05, that satisfied ${P}_k<\frac{\alpha }{m+1-k}$ were considered significant (see Supplementary Table 2).

## Results

### Hardy-Weinberg equilibrium

For phase I, we included 252 inpatients that received either amisulpride or olanzapine. For the entire trial that lasted 8 weeks, we included 212 individuals (‘completers’) that were treated with either only olanzapine or amisulpride or in case of a switch with both drugs consecutively. We analyzed the influence of the rs17782313 polymorphism on absolute and relative gain in body weight and absolute BMI increase. The demographic and clinical data can be found in Table 1. The rs17782313 genotype frequencies were in all cases determined successfully. The distribution of the observed genotype was in Hardy-Weinberg equilibrium for those in phase I (regardless of medication: χ = 0.604, *P* = 0.739; amisulpride only: χ = 1.626, *P* = 0.443; olanzapine only: χ = 0.330; *P* = 0.848; first episode: χ = 5.628; *P* = 0.060, one expected cell frequency was below 5) and those completing the entire 8 weeks (regardless of medication: χ = 0.593, *P* = 0.743; amisulpride only: χ = 1.100, *P* = 0.577, one expected cell frequency was below 5; olanzapine only: χ = 4.287, *P* = 0.117, one expected cell frequency was below 5; first episode: χ = 3.334; *P* = 0.189, one expected cell frequency was below 5).

**Table 1:** Baseline characteristics

Medication	Both antipsychotics	Amisulpride only	Olanzapine only
Phase I only/Entire trial (completers)			
Participants (*n*)^a^	252/212	129/85	123/75
Male (% of all participants)	52/50	53/57	51/44
Mean age (years)	41.5±11.4/41.7±11.4	42.3±11.8/42.4±12.3	40.7±11.1/41.1±11.6
Baseline weight (kg)	75.19±16.42/75.41±16.53	75.27±16.78/76.71±17.44	75.10±16.11/76.37±17.24
Baseline BMI (kg/m^2^)^a^	25.78±5.40/25.98±5.45	25.98±5.36/26.25±5.63	25.59±5.45/26.30±5.76
Caucasian descent (% of all participants)	97.6/97.2	96.9/96.5	98.4/98.7
First episode (*n*)	37/29	15/12	22/13
Antipsychotic Medication^b^			
Amisulpride only (*n*)	129/85	129/85	-/-
Olanzapine only, (*n*)	123/75	-/-	123/75
Amisulpride-Olanzapine switch (*n*)	-/25	-/-	-/-
Olanzapine-Amisulpride switch (*n*)	-/27	-/-	-/-

^a^The baseline height was missing for one individual, therefore the analyses regarding BMI include one patient less

^b^40 of the 252 participants that finished phase I dropped out while in phase II. This explains the missing participants in the second part of this table. In phase I, 129 patients received amisulpride, in phase II 85 of these stuck to amisulpride, 25 switched to olanzapine, 19 dropped out. In phase I, 123 patients received olanzapine, in phase II 75 of these stuck to olanzapine, 27 switched to amisulpride, 21 dropped out

### Baseline characteristics

The average baseline BMI ± SD (standard deviation) of all 252 individuals participating in phase I was 25.8±5.4 kg/m^2^ (24.6±4.2 kg/m^2^ for male participants and 27.0±6.2 kg/m^2^ for female participants). One hundred twenty-nine patients receiving amisulpride during that time span had an average baseline BMI ± SD of 26.0±5.4 kg/m^2^ (25.2±4.8 kg/m^2^ for males and 26.9±5.8 kg/m^2^ for females) while 123 individuals receiving olanzapine in the first 2 weeks had an average baseline BMI ± SD of 25.6±5.4 kg/m^2^ (24.0±3.5 kg/m^2^ for males and 27.2±6.6 kg/m^2^ for females). The average age of all 252 patients was 41.5 years and 52% were males, 37 individuals (14.7%) had their first episode. At baseline, there was a statistically significant difference in body weight in respect to the rs17782313 genotype in all individuals participating in phase I. TT carriers weighed 73.5 kg compared to 79.1 kg in TC carriers and 68.8 kg in CC carriers. The same could be observed in patients treated with amisulpride yet not olanzapine, though in all three groups TC carriers had the highest baseline body weight and CC carriers the lowest (see Table 2).

**Table 2:** Analysis of variance for the rs17782313 polymorphism for phase i and the entire trial

Both antipsychotics		TT *n*^a^ = 155/130	TC *n*^a^ = 84/70	CC *n*^a^ = 13/12	P*	P**
Phase I only	m_0_ (kg)	73.53±16.04	79.08±17.28	68.76±9.22	**0.021**	0.077
	m_2_ (kg)	74.32±15.74	79.85±17.00	70.14±10.39	**0.016**	**0.043**
	Δm_2-0_ (kg)	0.79±1.90	0.77±2.85	1.38±2.44	0.670	0.823
	relΔm_2-0_ (%)	1.23±2.68	1.10±3.65	1.92±3.37	0.637	0.823
	BMI_0_ (kg/m^2^)	25.47±5.45	26.47±5.48	25.09±3.84	0.360	0.192
	BMI_2_ (kg/m^2^)	25.74±5.34	26.71±5.33	25.56±3.93	0.330	0.167
	ΔBMI_2-0_ (kg/m^2^)	0.27±0.65	0.25±0.93	0.47±0.79	0.575	0.839
Entire trial^b^	m_0_ (kg)	74.26±16.53	78.75±16.96	68.32±9.48	0.059	0.275
	m_8_ (kg)	75.49±16.18	81.09±16.83	72.23±13.95	**0.039**	0.117
	Δm_8-0_ (kg)	1.23±3.08	2.34±4.00	3.91±6.58	0.174	0.063
	relΔm_8-0_ (%)	1.89±4.23	3.23±5.24	5.37±8.10	0.256	0.112
	BMI_0_ (kg/m^2^)	25.90±5.64	26.36±5.39	24.64±3.64	0.643	0.682
	BMI_8_ (kg/m^2^)	26.30±5.44	27.12±5.17	25.93±4.13	0.438	0.308
	ΔBMI_8-0_ (kg/m^2^)	0.40±1.05	0.75±1.30	1.29±1.99	0.179	0.072
Amisulpride only		TT n^a^=83/55	TC n^a^=41/26	CC n^a^=5/4	*P*	P^*^
Phase I only	m_0_ (kg)	72.68±16.72	81.56±16.11	66.66±6.79	**0.008**	**0.011**
	m_2_ (kg)	73.27±16.43	82.09±15.47	68.24±5.60	**0.005**	**0.007**
	Δm_2-0_ (kg)	0.59±1.75	0.53±2.62	1.58±2.13	0.407	0.738
	relΔm_2-0_ (%)	0.95±2.48	0.85±3.42	2.58±3.74	0.440	0.883
	BMI_0_ (kg/m^2^)	25.39±5.57	27.28±4.93	25.06±3.85	0.121	0.060
	BMI_2_ (kg/m^2^)	25.59±5.43	27.46±4.64	25.63±3.43	0.086	**0.036**
	ΔBMI_2-0_ (kg/m^2^)	0.20±0.60	0.17±0.86	0.57±0.73	0.390	0.824
Entire trial^b^	m_0_ (kg)	75.88±18.07	80.29±16.57	64.80±6.20	0.216	0.557
	m_8_ (kg)	76.57±17.03	82.57±16.01	68.40±4.06	0.156	0.278
	Δm_8-0_ (kg)	0.69±2.96	2.28±4.16	3.60±4.22	0.063	**0.043**
	relΔm_8-0_ (%)	1.33±4.24	3.11±5.14	5.97±7.48	0.177	0.103
	BMI_0_ (kg/m^2^)	26.30±6.19	26.52±4.67	23.71±2.77	0.650	0.974
	BMI_8_ (kg/m^2^)	26.52±5.75	27.25±4.28	24.98±1.55	0.681	0.696
	ΔBMI_8-0_ (kg/m^2^)	0.22±1.02	0.72±1.33	1.27±1.44	0.060	**0.028**
Olanzapine only		TT n^a^=72/43	TC n^a^=43/24	CC n^a^=8/8	*P*	P^*^
Phase I only	m_0_ (kg)	74.51±15.27	76.71±18.19	70.08±10.68	0.534	0.967
	m_2_ (kg)	75.53±14.92	77.70±18.26	71.33±12.76	0.545	0.692
	Δm_2-0_ (kg)	1.03±2.05	0.99±3.07	1.25±2.75	0.997	0.936
	relΔm_2-0_ (%)	1.57±2.86	1.33±3.88	1.50±3.32	0.964	0.798
	BMI_0_ (kg/m^2^)	25.57±5.35	25.68±5.91	25.11±4.10	0.985	0.921
	BMI_2_ (kg/m^2^)	25.92±5.25	26.00±5.87	25.51±4.45	0.967	0.801
	ΔBMI_2-0_ (kg/m^2^)	0.36±0.71	0.32±1.00	0.41±0.87	0.989	0.945
Entire trial^b^	m_0_ (kg)	75.76±16.03	79.57±20.67	70.08±10.68	0.383	0.910
	m_8_ (kg)	77.55±15.76	82.56±21.13	74.14±16.92	0.486	0.949
	Δm_8-0_ (kg)	1.79±3.13	2.99±4.39	4.06±7.77	0.676	0.495
	relΔm_8-0_ (%)	2.60±3.93	3.92±5.80	5.08±8.88	0.791	0.566
	BMI_0_ (kg/m^2^)	26.61±5.75	26.16±6.36	25.11±4.10	0.716	0.419
	BMI_8_ (kg/m^2^)	27.21±5.69	27.13±6.46	26.41±5.00	0.811	0.567
	ΔBMI_8-0_ (kg/m^2^)	0.60±1.09	0.97±1.39	1.30±2.31	0.754	0.544

*P-values for the comparison of TT carriers with TC carriers with CC carriers **P-values for the comparison of C allele carriers with TT carriers, ^a^number of carriers of each genotype for phase I/the entire trial, ^b^Baseline weight and BMI are given for patients finishing phase I and the entire trial separately, P-values in bold are significant

m_0_ baseline weight, m_n_ weight after n weeks, Δm_n-0_ weight gain after n weeks, relΔm_n-0_ relative weight gain after n weeks compared to baseline in %, BMI_0_ baseline BMI, BMI_n_ BMI after n weeks, ΔBMI_n-0_ BMI gain after n weeks

Those individuals completing the entire 8 weeks of the trial (‘completers’) numbered 212 and were on average 41.7 years of age. Baseline BMI and body weight almost did not differ from those finishing at least phase I. While it could be again observed that TC carriers had the highest baseline weight and CC carriers the lowest, there was no statistical significance (see Tables 1 and 2). First episode patients were on average 32.5 years old and had a baseline body weight of 72.3±12.2 kg (*P* = 0.285 compared to patients who did not have a first episode). TT-carriers weighed 68.9±9.8 kg, TC-carriers had a baseline weight of 74.0±13.2 kg, and the only CC homozygous participant had a baseline weight of 90.0 kg (see supplementary Tables 3 and 4).

### Weight gain in phase I

**Fig. 1:**
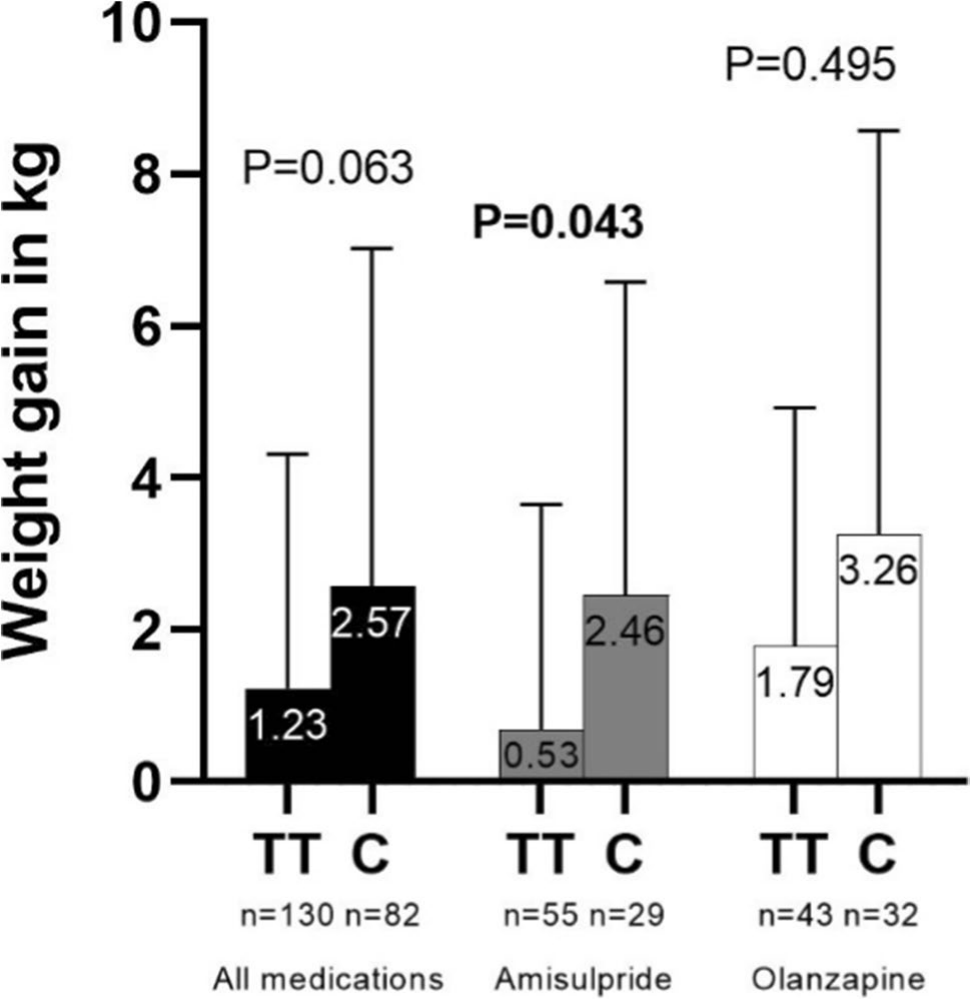
Weight gain in kg observed within 8 weeks of treatment depending on medication in each genotype. Data are presented as mean+SD. Statistical tests compare TT with C carriers

After 2 weeks of treatment, *n* = 130 males gained on average 0.8±2.6 kg and *n* = 122 females gained on average 0.8±2.0 kg (combined 0.8±2.3 kg). Patients homozygous for the T allele gained 0.8±1.9 kg, TC carriers gained 0.8±2.9 kg, and CC carriers gained 1.4±2.4 kg (*P* = 0.670). In those treated with amisulpride, TT carriers gained 0.6±1.8 kg, TC carriers gained 0.5±2.6 kg, and homozygous C carriers had an average weight gain of 1.6±2.1 kg (*P* = 0.407). Compared to that, patients receiving olanzapine had an average weight gain of 1.0±2.0 kg in TT, 1.0±3.1 kg in TC and 1.3±2.7 kg in CC (*P* = 0.997) (see Table 2 and Fig. 1). Regarding phase I, stepwise multiple linear regressions with relative weight gain in phase I as dependent variable and MC4R genotype, age, sex, baseline body weight, and smoking status did not show a significant association of the MC4R genotype with relative weight in all three medication groups and in those having their first episode of illness (0.343<P<0.912). In patients who had their first episode, TT carriers gained on average 1.9±1.9 kg, TC carriers gained 1.7±3.1 kg and the only homozygous C carrier gained 7 kg (see supplementary Table 4).

### Weight gain in ‘completers’

After 8 weeks of treatment, the *n* = 106 male subjects had a mean ± SD weight gain of 2.4±4.2 kg and the *n* = 106 female subjects gained 1.1±3.1 kg (combined 1.7±3.7 kg). Homozygous carriers of the T allele gained on average 1.2±3.1 kg compared to 2.3±4.0 kg in TC carriers and 3.9±6.6 kg in CC carriers (*P* = 0.174). Overall, C allele carriers (CC and TC) gained an average of 2.6±4.5 kg (*P* = 0.063 compared to TT-genotype). In those treated with amisulpride only during the entire trial, TT carriers gained 0.7±3.0 kg, TC carriers gained 2.3±4.2 kg, and CC carriers 3.6±4.2 kg (*P* = 0.063). When comparing those with TT genotype to C allele carriers, the difference in weight gain (0.7±3.0 kg vs. 2.5±4.1 kg) and in BMI gain (0.2±1.1 kg/m^2^ vs. 0.8±1.4 kg/m^2^) reached statistical significance (*P* = 0.043 respectively *P* = 0.028). Individuals that received exclusively olanzapine experienced an average increase in body weight of 1.7±3.1 kg (TT), 3.0±4.4 kg (TC) and 4.0±7.8 kg (CC), *P* = 0.676 (Table 2 and Fig. 1). We then conducted stepwise multiple linear regressions with relative weight gain over the entire 8 weeks as dependent variable and *MC4R* genotype, age, sex, baseline body weight, and smoking status as possible independent variables. In all ‘completers’ regardless of medication, we identified lower age (β = −0.191; *P* = 0.004), male sex (β = 0.193; *P* = 0.004), lower baseline body weight (β = −0.233; *P* < 0.001), non-smoking status (β = 0.107; *P* = 0.110), and the C-*MC4R* allele (β = 0.201; *P* = 0.002) as relevant factors. The overall model was statistically significant, F(5, 206) = 8.244, *P* < 0.001. Its *R*^2^ was 0.167 (adjusted *R*^2^ = 0.147). In patients receiving only amisulpride during the entire trial, we again identified lower age (β = −0.198; *P* = 0.049), male sex (β = 0.250; *P* = 0.015), lower baseline body weight (β = −0.408; *P* < 0.001), non-smoking status (β = 0.059; *P* = 0.529), and the C allele of *MC4R* (β = 0.244; *P* = 0.012) as relevant factors. The overall model was statistically significant, F(5, 79) = 7.372, *P* < 0.001. Its *R*^2^ was 0.318 (adjusted *R*^2^ = 0.275). In patients treated exclusively with olanzapine during the entire 8 weeks, no significant factor could be identified (*P* > 0.129, *MC4R*: β = 0.169; *P* = 0.144), the overall model lacked significance (*P* = 0.199). Its *R*^2^ would have been 0.098, its adjusted *R*^2^ 0.033. In first episode patients, TT carriers gained on average 2.8±2.6 kg compared to 3.8±3.4 kg in TC carriers. The patient with CC genotype gained 22 kg (*P* < 0.001 respectively *P* = 0.110 for the comparison of TT with C, see supplementary Table 4). A likewise conducted stepwise multiple linear regression analysis was not significant in first episode patients (overall model *P* = 0.235, C allele of MC4R *P* = 0.077).

### Multiple comparisons

Twenty-five variables were corrected for multiple comparisons by Holm’s method. The two lowest *p* values remained significant, these were the analysis of variance of weight gain within 8 weeks in first episode patients (*P* < 0.001, corrected α = 0.002) and the MC4R genotype as a factor in the stepwise multiple linear regression analysis of relative weight gain within 8 weeks in all completers (*P* = 0.0019, corrected α = 0.0021) (see supplementary Table 2).

## Discussion

We observed a statistically significant association between the rs17782313 polymorphism and absolute weight gain and absolute BMI gain after 8 weeks of antipsychotic treatment in the amisulpride subpopulation when comparing carriers of the C allele with TT carriers. The same direction of effect could be seen in the entire population and the olanzapine group, though it failed to reach conventional levels of statistical significance. The analysis indicates that the C allele might lead to a higher weight and BMI gain under SGA treatment.

TC carriers showed a higher baseline body weight and BMI than TT carriers in all populations, as previously shown in the GWAS (Loos et al. 2008) and in other studies (Beckers et al. 2011; Huang et al. 2011; Xi et al. 2012). The homozygotes for the C allele had a lower baseline weight and BMI in all subpopulations except first episode patients, which is contrary to earlier publications. This, however, might be explained by the low number of subjects compared to the other genotypes (*n* = 13 in the whole population, *n* = 5 in the amisulpride group, *n* = 8 in the olanzapine group and *n* = 1 in first episode patients). In addition, the high percentage of pretreated patients might have played a role in this, as only 14.7% of participants had their first episode.

Patients in the olanzapine medication group gained a mean ± SD body weight of 2.4±4.2 kg within 8 weeks. A meta-analysis from Allison et al. (1999) used a fixed-effects model to calculate an estimated weight change (kg) of 3.51 with a 95% confidence interval (3.29–3.73) after 10 weeks of treatment. Although there is no information on the percentage of drug-naïve patients and other demographic characteristics such as sex, baseline weight and age while the number of pretreated subjects in our study was rather high, these numbers are of comparable size. In the amisulpride subpopulation, a mean ± SD weight gain of 1.3± 3.5 kg occurred. Leucht et al. (2004) investigated the weight gain caused by treatment with amisulpride in a meta-analysis. Their estimate after 10 weeks of treatment was 0.80 kg, 95% CI (0.48–1.18) and therefore similar to the weight gain measured in our investigation as well. We observed that olanzapine caused a greater increase in body weight than amisulpride in a double-blind setting. The higher AIWG in patients treated with olanzapine is expected to be due to its affinity to the H1 (histamine 1) receptor and 5-HT2C (serotonin 2C) receptor in contrast to amisulpride binding the D2 and D3 (dopamine 3) receptor (Balt et al. 2011).

In first episode patients, the rs17782313 was significantly associated with weight gain and relative weight gain (compared to baseline) after 8 weeks, even after comparing for multiple corrections, though due to the low minor allele frequency only one individual was homozygous for the C-allele, and that patient happened to experience a multiple times larger increase in body weight. Czerwensky et al. (2013) analyzed a first episode subpopulation and discovered a significant association between the rs17782313 and AIWG. However, they reported 9 CC genotypes in 96 first episode patients. Zhang et al. (2016) on the other hand failed to reproduce these findings in their larger first episode population (n = 567, n_CC_ = 14). As schizophrenia is a chronic mental illness with long courses of disease, researchers might struggle to generate large samples of first episode patients, thus adversely affecting transferability of these results.

Patients homozygous for the rs17782313 C allele gained both more absolute and more relative weight than those heterozygous for the C allele or TT carriers in all three medication groups after 2 weeks as well as after 8 weeks of antipsychotic treatment. Despite these differences not reaching significant levels, a correction for known confounders in stepwise multiple linear regressions showed significant *p*-values in the whole study population and the amisulpride subpopulation for a time span of 8 weeks. We could reproduce the positive association of low baseline body weight, non-smoking and younger age with relative weight gain, which previous studies have also reported (Czerwensky et al. 2013; Gebhardt et al. 2009; Müller and Kennedy 2006). While sex differences in SGA-related weight gain still remain unclear (Gebhardt et al. 2009), our model showed a positive association with the male sex. As potential differences in gene expression related to AIWG between the sexes (Sainz et al. 2019) have been shown, further studies are required to clarify the influence of sex on antipsychotic-induced weight gain. Our regression model had a coefficient of determination *R*^2^ of 0.167, indicating that 16.7% of the percent weight variation in all patients can be explained by the factors of baseline weight, age, sex, smoking status, and the *MC4R* genotype.

As the increased weight gain is attributed to the C allele, the comparison of subjects homozygous or heterozygous for the C allele to those homozygous for the T allele showed a tendency in the whole population and in the amisulpride subpopulation of a significant difference for the entire duration of the trial but not after 2 weeks. Such short intervals might be simply not long enough for the genetic influence on AIWG to be seen. Czerwensky et al. (2013) found their significant association after 4 weeks of treatment, Chowdhury et al. (2013) saw tendencies in certain groups of patients after 6 weeks, and Zhang et al. (2019) could not find a significant correlation after 6 weeks. Temporal dynamics of a possible connection of the rs17782313 and AIWG have not been described so far, and our results in this regard should be interpreted with caution as not all patients participating in phase I finished the entire trial. Furthermore, a ‘ceiling effect’ in patients treated with olanzapine could have played a role here (Kinon et al. 2005).

When comparing the two antipsychotic substances, in patients only treated with amisulpride, the relative weight gain of the CC allele carriers after the full 8 weeks of treatment was 4.5 times higher and of the TC allele carriers 2.3 times higher than that of TT carriers, yet in patients only treated with olanzapine, it was 2.0 times higher in CC carriers respectively 1.5 times higher in heterozygous patients than in those homozygous for the T allele. Furthermore, in the stepwise multiple linear regressions, the influence of the *MC4R* genotype on relative weight gain was stronger in patients treated with amisulpride than olanzapine (β = 0.244; *P* = 0.012 vs. β = 0.169; *P* = 0.144). This might suggest that the rs17782313 polymorphism has different capabilities of enhancing weight gain under SGA treatment depending on the substance given, or rather the interallelic differences in weight gain may depend on the substance. The rs17782313 polymorphism is expected to result in some loss of function of the *MC4R* (human melanocortin 4 receptor), which would normally lower food intake when stimulated (Balt et al. 2011; Fan and Tao 2009). Therefore, the presence of a C allele would increase energy intake and result in a higher body weight. As mentioned above, olanzapine binds to the H_1_ receptor and 5-HT_2C_ receptor. Antagonism to the latter results in decreased levels of α-MSH (α-melanocyte stimulating hormone) (Balt et al. 2011), which is the endogenous, stimulating ligand of MC4R. Lower levels of α-MSH therefore should result in a higher food intake. Through this mechanism, treatment with olanzapine might induce weight gain in all three rs17782313 genotypes and show an additive effect in C allele carriers, as proposed by others (Czerwensky et al. 2013). Amisulpride, on the other hand, is not considered to influence the α-MSH level. When treated with amisulpride, the α-MSH level remains unaffected and therefore does not cause extra weight gain in all allele carriers. The additional increase in body weight under treatment with olanzapine in all genotypes through this pathway might ‘conceal’, so to speak, the effect caused by rs17782313 and flatten relative, interallelic differences (Table 2). However, these results must be interpreted carefully as the number of patients homozygous for the C allele was small (for the complete trial *n* = 4 in the amisulpride subpopulation and *n* = 8 in the olanzapine subpopulation) and further research is needed to support this hypothesis.

We decided to perform Holm’s procedure to correct for multiple comparisons to evaluate robustness of our statistical findings. The MC4R genotype remained significantly associated with weight gain after 8 weeks in first episode patients (TT vs TC vs CC) and as a factor in multiple linear regression in ‘completers’ of the trial. Therefore, our statistical findings should be interpreted carefully. However, we believe that exploratory approaches do not provide strict evidence for certain genetic effects in the first place, but they help investigate possible associations and are necessary for later more rigorous studies. Moreover, even results that have no significant association with a proposed effect but show a tendency should be reported as they would otherwise lead to publication bias.

Some limitations of this study must be mentioned. First, although there were only two different SGAs given and the number of subjects treated with each one is therefore relatively large, the overall study population consisted of only 252 patients for the first 2 weeks and 212 individuals for the entire 8 weeks and is smaller than in the previous analyses (Chowdhury et al. 2013; Czerwensky et al. 2013; Zhang et al. 2019). However, these previous trials included naturalistic samples of patients while the SWITCH study was a multicenter double blinded, controlled study, which makes this analysis to date unique. Second, the number of pretreated patients was rather high, as only 14.7% had their first episode. Nevertheless, as schizophrenia is a chronic disease and many patients receive treatment for long periods of time, the effect in premedicated patients does not necessarily lack relevance for clinical settings. Third, the influence of comedication cannot be fully measured. Some drugs, especially antidepressants, can induce weight gain on their own (Drieling et al. 2005). Nonetheless, the comedication in this study was quite clear, patients received only one SGA, and certain drugs as mood stabilizers or recently initiated antidepressants were not to be given. Third, the duration of this trial was only 8 weeks and some patients dropped out early, which may negatively impact the generalizability of our findings, though previous studies analyzing the influence of the rs17782313 on AIWG were all shorter. In addition to that, the bulk of AIWG associated with olanzapine occurs within the first 12 weeks of treatment (Kinon et al. 2005) and in the case of clozapine, with the patients in the SWITCH study unfortunately not received, within 6 weeks even in pretreated patients (Meltzer et al. 2003). Future investigations on genetic influence on olanzapine-associated weight gain and clozapine-associated weight gain should take those time frames into account. Finally, there is still the possibility that neither the MC4R gene nor the rs17782313 single nucleotide polymorphism have an influence on AIWG at all, but our findings are coincidental. As AIWG most likely has a polygenic nature (Zhang et al. 2016), our candidate gene approach cannot prove a causal link between the SNP and AIWG but show statistically significant association, contributing to the existing and future studies and providing data for meta analyses.

In this study, we report a significant association of amisulpride-induced weight gain after 8 weeks of treatment and the rs17782313 C allele, a polymorphism located in the promoter region of *MC4R*, in a predominantly Caucasian population and observed a tendency in the overall and olanzapine-treated population. Our findings support the earlier assumption, namely that the rs17782313 polymorphism might play a role in SGA-related weight gain, yet fail to remain significant when corrected for multiple comparisons. To our knowledge, our explanation involving the *MC4R* for relative, interallelic differences depending on the medication given has not been given so far. Overall, further studies with larger populations are needed to clarify the influence of the rs17782313 polymorphism on SGA-related weight gain, investigate differences regarding different antipsychotics, and set a basis for future meta-analyses.

## Supplementary information

Supplementary Table 1 Variables and Normal Distribution. Supplementary Table 2 Holm’s Test for Multiple Comparisons. Supplementary Table 3 Baseline Characteristics of Patients With a First Episode. Supplementary Table 4 Analysis of Variance for the rs17782313 Polymorphism for Phase I and the Entire Trial in Patients With a First Episode (PDF 201 kb)

## Abbreviations

5-HT_2C_: serotonin 2C receptor
α-MSH: α-melanocyte stimulating hormone
AIWG: antipsychotic-induced weight gain
ANOVA: analysis of variance
BMI: body mass index
D_2_: dopamine 2 receptor
D_3_: dopamine 3 receptor
GWAS: genome wide association study
H_1_: histamine 1 receptor
MC4R: human melanocortin 4 receptor
*MC4R*: human melanocortin 4 receptor gene
SD: standard deviation
SGA: second-generation antipsychotic
SNP: single nucleotide polymorphism
