# Supplementary material for: Genetic association of the rs17782313 polymorphism with antipsychotic-induced weight gain
Source: Psychopharmacology (Berl). 2023 Feb 9;240(4):899–908. doi: 10.1007/s00213-023-06331-9 (PMC10006246; doi:10.1007/s00213-023-06331-9)
Supplement: Supplementary file 1 — Variables and Normal Distribution. Supplementary Table 2 Holm’s Test for Multiple Comparisons. Supplementary Table 3 Baseline Characteristics of Patients With a First Episode. Supplementary Table 4 Analysis of Variance for the rs17782313 Polymorphism for Phase I and the Entire Trial in Patients With a First Episode (PDF 201 kb) [file 213_2023_6331_MOESM1_ESM.pdf]

Article title: Genetic Association of the rs17782313 Polymorphism with Antipsychotic Induced Weight Gain

Journal name: Psychopharmacology

Authors: Korbinian Felix Schreyer[1], Stefan Leucht[2], Stephan Heres[3], Werner Steimer[1]

Affiliation: 1 Department of Clinical Chemistry and Pathobiochemistry, Technical University of Munich, Department of Medicine, Ismaninger Str. 22, 81675 Munich, Germany
2 Department of Psychiatry and Psychotherapy, Technical University of Munich, Department of Medicine, Ismaninger Str. 22, 81675 Munich, Germany
3 kbo-Klinik für Psychiatrie und Psychotherapie Schwabing, Kölner Platz 1, 80804 Munich, Germany

Corresponding author: korbinian.schreyer@tum.de

Supplementary Table 1 Variables and Normal Distribution

Medication Group/Phase	Variable	P*	P**
Both Antipsychotics/Phase I	m_0 (kg)	X	X
	m_2 (kg)	X	N
	$\Delta m_{2\text{-}0}$ (kg)	X	X
	rel$\Delta m_{2\text{-}0}$ (%)	X	X
	BMI_0 (kg/m²)	X	X
	BMI_2 (kg/m²)	X	X
	$\Delta BMI_{2\text{-}0}$ (kg/m²)	X	X
Both Antipsychotics/Entire Trial	m_0 (kg)	X	X
	m_8 (kg)	X	X
	$\Delta m_{8\text{-}0}$ (kg)	X	X
	rel$\Delta m_{8\text{-}0}$ (%)	X	X
	BMI_0 (kg/m²)	X	X
	BMI_8 (kg/m²)	X	X
	$\Delta BMI_{8\text{-}0}$ (kg/m²)	X	X
Olanzapine Only/Phase I	m_0 (kg)	X	X
	m_2 (kg)	N	N
	$\Delta m_{2\text{-}0}$ (kg)	X	X
	rel$\Delta m_{2\text{-}0}$ (%)	X	X
	BMI_0 (kg/m²)	X	X
	BMI_2 (kg/m²)	X	X
	$\Delta BMI_{2\text{-}0}$ (kg/m²)	X	X
Olanzapine Only/Entire Trial	m_0 (kg)	N	X
	m_8 (kg)	X	X
	$\Delta m_{8\text{-}0}$ (kg)	X	X
	rel$\Delta m_{8\text{-}0}$ (%)	X	X
	BMI_0 (kg/m²)	X	X
	BMI_8 (kg/m²)	X	X
	$\Delta BMI_{8\text{-}0}$ (kg/m²)	X	X
Amisulpride Only/Phase I	m_0 (kg)	X	X
	m_2 (kg)	X	X
	$\Delta m_{2\text{-}0}$ (kg)	X	X
	rel$\Delta m_{2\text{-}0}$ (%)	X	X
	BMI_0 (kg/m²)	X	X
	BMI_2 (kg/m²)	X	X
	$\Delta BMI_{2\text{-}0}$ (kg/m²)	X	X
Amisulpride Only/Entire Trial	m_0 (kg)	N	X
	m_8 (kg)	N	N
	$\Delta m_{8\text{-}0}$ (kg)	N	N
	rel$\Delta m_{8\text{-}0}$ (%)	X	X
	BMI_0 (kg/m²)	N	X
	BMI_8 (kg/m²)	N	X
	$\Delta BMI_{8\text{-}0}$ (kg/m²)	N	X
First Episode/Phase I	m_0 (kg)	N	N
	m_2 (kg)	N	N
	$\Delta m_{2\text{-}0}$ (kg)	N	N
	rel$\Delta m_{2\text{-}0}$ (%)	N	N
	BMI_0 (kg/m²)	X	X
	BMI_2 (kg/m²)	N	N
	$\Delta BMI_{2\text{-}0}$ (kg/m²)	N	N
First Episode/Entire Trial	m_0 (kg)	N	N
	m_8 (kg)	N	N
	$\Delta m_{8\text{-}0}$ (kg)	N	X
	rel$\Delta m_{8\text{-}0}$ (%)	N	N
	BMI_0 (kg/m²)	N	N
	BMI_8 (kg/m²)	N	N
	$\Delta BMI_{8\text{-}0}$ (kg/m²)	N	X
[a]Comparison of Baseline Weight First Episode / Not First Episode	m_0 (kg) First Episode	X	
	m_0 (kg) Not First Episode	X	
	P*: TT, TC, and CC; P**: TT and C. N: Normal distribution. X: No normal distribution		
	[a]No genotype subgroups in these variables		

**Supplementary Table 2** Holm's Test for Multiple Comparisons		
P-Value P_k	Index k	$\frac{\alpha}{m+1-k}$
**0,000009**[a]	**1**	**0,002**
**0.0019**[b]	**2**	**0,00208333**
0.012	3	0,00217391
0.043	4	0,00227273
0.063	5	0,00238095
0.063	6	0,0025
0.077	7	0,00263158
0.110	8	0,00277778
0.144	9	0,00294118
0.161	10	0,003125
0.174	11	0,00333333
0.344	12	0,00357143
0.407	13	0,00384615
0.495	14	0,00416667
0.638	15	0,00454545
0.670	16	0,005
0.676	17	0,00555556
0.738	18	0,00625
0.823	19	0,00714286
0.844	20	0,00833333
0.883	21	0,01
0.902	22	0,0125
0.912	23	0,01666667
0.936	24	0,025
0.997	25	0,05

[a]Δm8-0 (kg) TT vs TC vs CC in first episode patients, [b]MC4R genotype as factor in stepwise multiple linear regression in 'completers'

Supplementary Table 3 Baseline Characteristics of Patients With a First Episode

Medication	Phase I only	Entire trial (completers)
Participants (n)[a]	37	29
Male (% of all participants)	65	59
Mean age (years)	32.5±11.3	34.5±11.9
Baseline weight (kg)	72.26±12.17	71.80±11.94
Baseline BMI (kg/m²)[b]	23.38±2.89	23.32±2.81
Caucasian descent (% of all participants)	97.3	96.6
**Antipsychotic Medication**		
Amisulpride only (n)	15	12
Olanzapine only, (n)	22	13
Amisulpride-Olanzapine switch (n)	-	0
Olanzapine-Amisulpride switch (n)	-	4

[a]The baseline height was missing for one individual, therefore the analyses regarding BMI include one patient less

Supplementary Table 4 Analysis of Variance for the rs17782313 Polymorphism for Phase I and the Entire Trial in Patients With a First Episode

		TT n=16	TC n=20	CC n=1	P*	P**
Phase I only	m_0 (kg)	68.93±9.81	74.03±13.24	90.00	0.154	0.149
	m_2 (kg)	70.82±11.08	75.78±14.38	97.00	0.124	0.185
	Δm_{2-0} (kg)	1.89±1.86	1.75±3.08	7.00	0.161	0.902
	$rel\Delta m_{2-0}$ (%)	2.58±2.35	2.21±4.49	7.78	0.349	0.926
	BMI_0 (kg/m²)	21.86±1.67	24.38±3.17	26.01	**0.007**	**0.002**
	BMI_2 (kg/m²)	22.43±1.95	24.95±3.60	28.04	**0.030**	**0.007**
	ΔBMI_{2-0} (kg/m²)	0.57±0.55	0.57±1.04	2.02	0.267	0.823
		TT n=13	TC n=15	CC n=1	P*	P**
Entire trial[b]	m_0 (kg)	69.04±10.85	72.98±12.34	90.00	0.211	0.270
	m_8 (kg)	71.86±11.82	76.79±13.80	112.00	**0.020**	0.192
	Δm_{8-0} (kg)	2.82±2.58	3.81±3.35	22.00	**<0.001**	0.110
	$rel\Delta m_{8-0}$ (%)	4.04±3.57	5.12±4.77	24.44	**<0.001**	0.276
	BMI_0 (kg/m²)	21.84±1.86	24.32±3.00	26.01	**0.040**	**0.013**
	BMI_8 (kg/m²)	22.65±2.09	25.56±3.33	32.37	0.071	**0.005**
	ΔBMI_{8-0} (kg/m²)	0.81±0.76	1.24±1.13	6.36	0.274	0.059

*P-values for the comparison of TT carriers with TC carriers with CC carriers **P-values for the comparison of C allele carriers with TT carriers, [a]number of carriers of each genotype for phase I / the entire trial, [b]Baseline weight and BMI are given for patients finishing phase I and the entire trial separately

m_0 baseline weight, m_n weight after n weeks, Δm_{n-0} weight gain after n weeks, $rel\Delta m_{n-0}$ relative weight gain after n weeks compared to baseline in %, BMI_0 baseline BMI, BMI_n BMI after n weeks, ΔBMI_{n-0} BMI gain after n weeks.

Relative weight gain and relative BMI increase compared to baseline are mathematically equal, thus the latter was not added to the table
